# Using Community Engagement to Inform and Implement a Community-Randomized Controlled Trial in the Anishinaabek Cervical Cancer Screening Study

**DOI:** 10.3389/fonc.2014.00027

**Published:** 2014-02-19

**Authors:** Brianne Wood, Ann N. Burchell, Nicholas Escott, Julian Little, Marion Maar, Gina Ogilvie, Alberto Severini, Lisa Bishop, Kyla Morrisseau, Ingeborg Zehbe

**Affiliations:** ^1^Thunder Bay Regional Research Institute, Thunder Bay, ON, Canada; ^2^Ontario HIV Treatment Network, Toronto, ON, Canada; ^3^Dalla Lana School of Public Health, Toronto, ON, Canada; ^4^Department of Pathology and Laboratory Medicine, Thunder Bay Regional Health Sciences Centre, Thunder Bay, ON, Canada; ^5^Department of Epidemiology and Community Medicine, University of Ottawa, Ottawa, ON, Canada; ^6^Faculty of Human Sciences, Northern Ontario School of Medicine, Sudbury, ON, Canada; ^7^Clinical Prevention Services, BC Centre for Disease Control, Vancouver, BC, Canada; ^8^Viral Exanthemata and STD Section, National Microbiology Laboratory, Public Health Agency of Canada, Winnipeg, MB, Canada; ^9^Biology Department, Lakehead University, Thunder Bay, ON, Canada

**Keywords:** indigenous health, women’s health, social determinants of health disparities, community engagement, cervical screening

## Abstract

Social, political, and economic factors are directly and indirectly associated with the quality and distribution of health resources across Canada. First Nations (FN) women in particular, endure a disproportionate burden of ill health in contrast to the mainstream population. The complex relationship of health, social, and historical determinants are inherent to increased cervical cancer in FN women. This can be traced back to the colonial oppression suffered by Canadian FN and the social inequalities they have since faced. Screening – the Papinacolaou (Pap) test – and early immunization have rendered cervical cancer almost entirely preventable but despite these options, FN women endure notably higher rates of diagnosis and mortality due to cervical cancer. The Anishinaabek Cervical Cancer Screening Study (ACCSS) is a participatory action research project investigating the factors underlying the cervical cancer burden in FN women. ACCSS is a collaboration with 11 FN communities in Northwest Ontario, Canada, and a multidisciplinary research team from across Canada with expertise in cancer biology, epidemiology, medical anthropology, public health, virology, women’s health, and pathology. Interviews with healthcare providers and community members revealed that prior to any formal data collection education must be offered. Consequently, an educational component was integrated into the existing quantitative design of the study: a two-armed, community-randomized trial that compares the uptake of two different cervical screening modalities. In ACCSS, the Research Team integrates community engagement and the flexible nature of participatory research with the scientific rigor of a randomized controlled trial. ACCSS findings will inform culturally appropriate screening strategies, aiming to reduce the disproportionate burden of cervical disease in concert with priorities of the partner FN communities.

## Introduction

Cervical cancer incidence and mortality has reduced dramatically since the introduction of cervical cancer screening using cervical cytology 50 years ago, particularly in developed nations (1). Internationally, vulnerable populations, including women in developing countries and immigrant and indigenous populations experience higher rates of cervical cancer compared to the general population (2–6). Such discrepancies are largely attributable to sub-optimal screening and inadequate follow-up of abnormal results. Though the data is limited, Canadian Aboriginal groups, who include First Nations (FN), the Métis, and Inuit, exhibit 1.7–3.5 times increased cervical cancer incidence, as well as increased prevalence of human papillomavirus (HPV) infection – the underlying cause of cervical cancer (2, 7, 8). Aboriginal women also suffer lower survival from cervical cancer in contrast to the general population (9–11). Understanding and addressing the factors that influence cervical screening access and preferences in Aboriginal women are crucial for reducing these existing health disparities.

Clinician administered Pap tests are currently the standard screening tests in Ontario, though strategies that target HPV are making their way into practice (12, 13). Recent studies have indicated that preventative measures like HPV vaccination and screening with HPV DNA testing may prevent related morbidities. HPV testing is superior to traditional cytology testing in sensitivity and reproducibility of results (13–16). HPV vaccination, testing, and surveillance programs will be integrated into the Ontario Cervical Screening Program over the next few years (12, 13, 16, 17). As new cervical screening programing is rolled out, we may need a tailored approach to best reach Aboriginal women.

The relationship between socio-economic disparities and poor health outcomes in Aboriginal populations is well-established in the literature, though poorly acknowledged in Canadian healthcare (18, 19). The implications of social, political, and historical contexts on the cervical cancer screening preferences in Aboriginal women remain unclear. Individual-level factors (e.g., living conditions and food security) and structural determinants (e.g., healthcare and educational resources and systems) interact with life experiences, particularly colonial experiences, social exclusion, and gender, to shape the health profiles of Aboriginal peoples [(20, 21); Maar et al., under review]. Specifically, social marginalization and colonial legacy have dramatically shaped the health of Aboriginal women [(21); Maar et al., under review; Wakewich et al. manuscript in progress]. For a female-specific condition like cervical cancer, we need to better understand how these inequities affect health care seeking behaviors and how to overcome these barriers. HPV testing of self-collected samples has been shown to be a desirable option for cervical screening by addressing barriers of personal comfort and access to health care, particularly among underserviced and lower socio-economic populations [(14, 15, 20, 22–25); Maar et al., under review]. However, the acceptability of self-sampling and any improvements to screening uptake in Aboriginal populations are still not known, despite suggested effectiveness in other high-risk groups. The Anishinaabek Cervical Cancer Screening Study (ACCSS) aims to fill this gap by assessing whether self-collected HPV testing is a sustainable and culturally appropriate cervical screening approach in 11 FN communities in Northwest Ontario, Canada. This study uses a mixed-methods approach that began with *qualitative* interviews and focus groups that helped shape the subsequent *quantitative* two-armed, community-randomized controlled screening trial.

Under a participatory action research (PAR) framework, ACCSS collaborates with partner FN communities to reflect on existing cervical screening knowledge, attitudes, and behaviors (KAB). This process complements the rigor of the subsequent cervical cancer screening trial (26, 27). ACCSS aims to identify the important factors of a culturally appropriate cervical screening program in FN communities through community-based research. In this paper, we describe the community engagement processes, the joint decision-making, and the subsequent execution of the community-randomized controlled trial (CRCT). At the time of submission, ACCSS had completed the qualitative data collection, the first phase of cervical screening in the 11 partner communities, and a reflection meeting was held with community-based research assistants (CBRAs) and the community health representatives who form the Community Steering Committee (CSC) to inform Phase 2 of the ACCSS trial.

## Materials and Methods

### Study design

**Figure 1:**
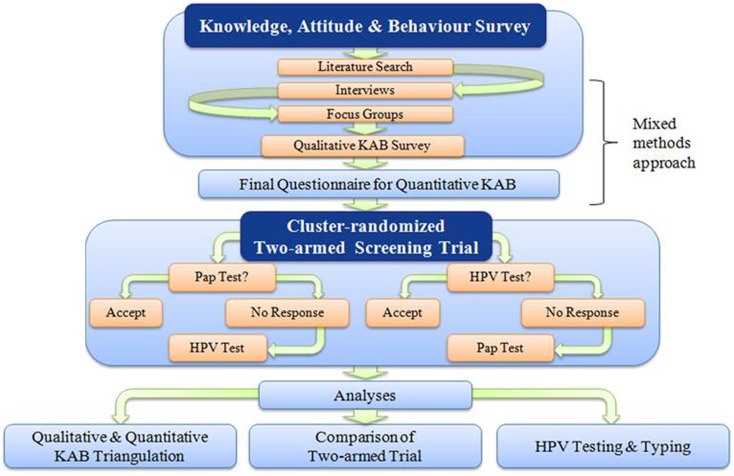
**A schematic representation of the Anishinaabek Cervical Cancer Screening Study**. This diagram reflects the action-feedback cycle that is integral to the PAR/CRCT framework.

The ACCSS is a mixed-methods research project that is taking place in 11 FN communities in the Robinson-Superior Treaty area of Northwest Ontario, Canada. The qualitative data collection of the project was completed in the first 2 years (e.g., the interviews and focus groups were completed between July 2011 and July 2012) following the signing of the Research Agreements with Chiefs and Councils of each community. This procedure and the following meet and greet visits to the community health centers are described in detail elsewhere [(28); Maar et al., under review]. Figure 1 illustrates how the multiple components interact and inform the proceeding actions of ACCSS.

Under a PAR framework, the ACCSS study design constantly adapts to new information provided through collaborative discussion with partner communities. The qualitative component of the study was structured to complement and inform the following quantitative portion of the study, including a questionnaire as part of a cervical screening intervention trial. During the qualitative portion, KAB related to cervical cancer screening were investigated through *key informant interviews* [methods described in Ref. (20)] with local healthcare provider and professionals, and *focus groups* (methods to be described in Wakewich et al., manuscript in progress) with community women. Input and reflection from community members, healthcare providers, and stakeholders is dynamically integrated into the design of the CRCT. Recruitment strategies and the offer of the cervical screening intervention are tailored to the unique social and political environments in each community, shaping the implementation of ACCSS in each FN community.

Qualitative results paired with input from the CSC and CBRAs informed the development of the epidemiological questionnaires of the screening trial. With this quantitative trial, we will assess whether screening participation increases when offering HPV testing based on self-sampling instead of Pap screening. In communities randomized to arm A, Pap cytology taken by a health professional is initially offered, and women who do not accept will then be offered HPV testing based on self-sampling in the second phase of the screening trial. In communities randomized to arm B, the reverse order is applied such that HPV self-testing is first offered, then the women who do not accept will then be offered Pap screening. We will measure whether self-sampling can increase screening attendance, and we will assess the psychosocial impact of the uptake of Pap testing compared to the uptake of HPV self-testing.

During the first offer of screening, CBRAs organize and facilitate educational events to recruit women into the CRCT, and later followed up with individual appointments to work through the informed consent, questionnaire, and offer of cervical screening with participants. After a 3-month period of screening, those who did not respond to the first offer of cervical screening are offered the reverse method of screening by the CBRAs.

At the completion of the first 3-month screening phase, the Research Team and community members took a 1- to-2-month reflection period as an opportunity to evaluate the methods and processes from the first round of screening, to inform the implementation of the second phase of the trial; the CBRAs will also use this opportunity to reconnect with participants from the first phase to conduct the follow-up questionnaire.

### FN partner communities

Community-based collaborative research is consistent with the aims of Aboriginal community self-determination and empowerment. The concept of *ethical space* helped to open dialog between the Research Team and community partners and has been described in detail elsewhere (28, 29). Community stakeholders and healthcare providers actively participate in the research process, contributing to the design and implementation of ACCSS, in addition to the interpretation and dissemination of the data.

### Study population

Since successfully completing our pilot study in the Fort William First Nation (30), we have established 10 additional partnerships with Robinson-Superior FN in Northwest Ontario, Canada (see Table 1) as evidenced by ratified Research Agreements. These communities, which are mostly located around the northern shore of Lake Superior and around Lake Nipigon were selected because of their geographic distribution, and because they represent a diverse range of political and economic processes, health care systems, social environments, and access to health and social services. In our partner communities and many communities in Northwest Ontario, Oji-Cree (also known as Severn Ojibwe) is a dialect of the Ojibwe and Cree languages, and a traditional language from our partner communities. “Anishinaabek” refers to “the people” in Oji-Cree.

**Table 1:** **Partner communities and their AANDC population statistics**.

Name of Robinson-Superior First Nation	Total registered females (as of January 2013)	Total registered on-reserve females (as of January 2013)
Animbiigo Zaagi’ing Anishinaabek (Lake Nipigon)^a^	236	2
Biinjitiwabik Zaaging Anishinaabek (Rocky Bay)	360	163
Bingwi Neyaashi Anishinaabek (Sand Point)^a^	113	34
Fort William First Nation	1093	460
Kiashke Zaaging Anishinaabek (Gull Bay)	600	149
Long Lake #58 First Nation	736	235
Pays Plat First Nation	107^b^	35^b^
Pic Mobert First Nation	451	157
6pt] Ojibways of Pic River	581	272
Red Rock First Nation (Lake	909	127
Helend)
Whitesand First Nation	590	154

*^a^Communities who are still working to secure a large land base for their reserve; consequently very few registered members actually live *on-reserve* (as per communication with the respective Health Director)*.

*^b^No sex-specific population estimates for Pays Plat. Of the 210 registered band members in January 2013, Statistics Canada Ontario population estimates suggest almost 52% of the Aboriginal population is female. Statistics Canada reported 35 on-reserve women in Pays Plat according to the last census (31)*.

**Figure 2:**
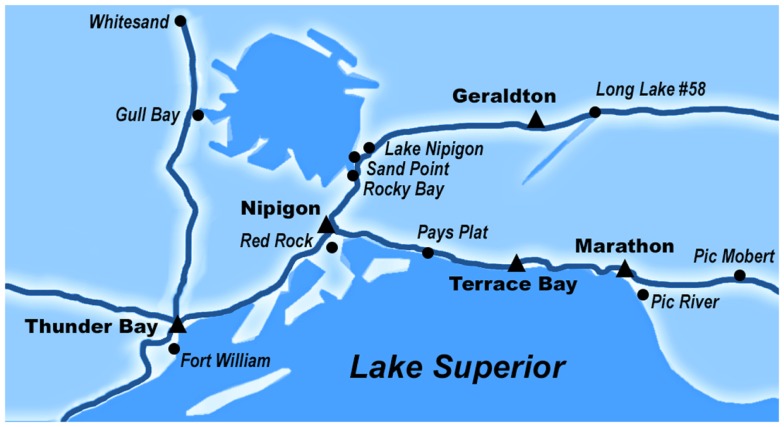
**The map of our partner communities of the Robinson-Superior Treaty in Northwestern Ontario, Canada**. Our partner communities are contained in the Thunder Bay District census area, which has a land area of 103,720 km^2^. All of our partner communities are accessible by road. Pic Mobert First Nation is the farthest distance away from the study center in Thunder Bay at approximately 360 km east of the city, and Fort William First Nation is adjacent to Thunder Bay.

The female membership in our partner communities ranged from 102 to 1096 (31, 32). Females between 25 and 69 years who are registered with 1 of the 11 FN communities (see Table 1) or live on-reserve (e.g., married to someone in the community) who have an Ontario Health Insurance Plan were invited to participate in the ACCSS CRCT. Women under 25 were excluded because high rates of transient HPV infections in this age group could result in a high false positive rate (33); women 70 years or older were excluded as a reflection of the low likelihood of incident high-grade abnormalities and in accordance with provincial guidelines (33–36). Though Ontario recommendations suggest that sexually active women should begin cervical screening at 21 years of age, the Canadian Task Force for Preventative Medicine recommends that routine cervical screening should begin at age 25 (36). Women who are currently pregnant are asked to take part after they give birth, and women with known complete hysterectomies are not invited to participate. Several Health Directors expressed concern that many of their band members live off-reserve, either temporarily or permanently, in the urban areas in the Robinson-Superior Treaty. Consequently, we have opened our study to women in the metropolitan areas of the Thunder Bay District (in which the Robinson-Superior Treaty is contained – see Figure 2), which broadens and diversifies our sample.

Table 1 depicts the population statistics from Aboriginal and Northern Development Canada (AANDC) for our 11 FN partner communities (30–32). These statistics represent female registered band members across all ages.

### Community steering committee

For PAR, we needed to expand our network to include partners who provide input on the research methodology and the cultural appropriateness of our cervical screening intervention strategy. We had already engaged the political leaders of the community – the Chiefs and Councils who had signed a Research Agreement – and we had made contact with the Health Directors and Health Representatives who provided interviews during the qualitative phase of ACCSS. After analyzing the interview and focus group documentation, we recognized that we needed to incorporate cervical screening education and awareness components into the CRCT design of ACCSS. We worked with Chiefs, Councils, and our respective community health contacts to identify two contacts from their community who were interested in partnering with ACCSS to implement the education as part of the trial.

Community health and social representatives attended a meeting with the Research Team to discuss effective health promotion and educational techniques for cervical screening (Zehbe et al. Manuscript in progress). Meeting attendees agreed to advise the Research Team on the design and implementation of the project in their community, establishing a CSC. CSC teleconferences occurred every 1 or 2 months from that meeting forward, or more frequently when more input was required (e.g., during the development of the pamphlet, the logo, and the questionnaires). The CSC was particularly focused on increasing awareness about cervical cancer and HPV within their communities, and ensuring that appropriate medical follow-up was offered to participants once the “research results” were obtained.

### Community-based research assistants

In collaboration with the CSC, we hired local and whenever possible Aboriginal CBRAs to implement the study in their community, acting as a link between the Research Team in Thunder Bay and their community. After coming to an agreement on a job description, CSC members posted the advertisements in the community and provided the Research Team with names of candidates for the position.

The CBRAs were hired in early 2013 after an in-person or phone interview with the Research Team. Some CSC members identified their preferred candidate for the CBRA position, though they left the ultimate decision to the Research Team. Some CSC members initially experienced difficulty finding local candidates who would be suitable when we first began searching for a CBRA. In some communities, women who were already employed at the on-reserve Health Center were recruited by the Health Director. In one community, an FN woman from a different community met with Health Directors to apply for the CBRA position when there were no local applicants. This CBRAs also suggested that we include off-reserve urban FN women in our recruitment strategy, which was agreed upon by the CSC and remaining CBRAs.

Most CBRAs were recruited within 3 months of our job offer posting, and they attended a welcome dinner and a full-day intensive training in Thunder Bay. At the training, CBRAs participated in a presentation about the biology and epidemiology of cervical cancer, led by the pathologist/colposcopist on the Research Team Dr. Nicholas Escott, and learned about ACCSS rationale, study design, and the research protocol through the principal investigator Dr. Ingeborg Zehbe and the research coordinator Brianne Wood. At the end of the day, deliverables that were required to implement the project were identified and delegated to the Research Team, the CBRAs, the CSC, and their political leaders.

The CBRAs returned to their communities with five primary objectives:

(i) Identify and recruit eligible women into the screening trial.
(ii) Interact with and assist the potential participants.
(iii) Administer the study questionnaires (i.e., baseline and follow-ups), and provide women with the opportunity to participate in cervical screening.
(iv) Communicate frequently with Research Team members (Brianne Wood, Ingeborg Zehbe) to provide updates and engage in discussions to guide future actions and improve study methodology.
(v) Continue to engage the CSC, Chiefs, and Council with ACCSS, and ensure constant communication between all community partners.

In partnership with the CSC, we eventually hired a CBRA for the community who did not find a candidate prior to the training, and held a one-on-one training session at the Research Team’s central office in Thunder Bay that mirrored the agenda of the group training. At the time of publication, four CBRAs had left their positions for personal reasons and new candidates were suggested by the local CSC representatives. These CBRA candidates were similarly interviewed, and then brought into the Research Team’s central office for one-on-one training.

After the training, CBRAs began to plan an educational event to launch the trial in their community, to the first phase of cervical screening offers in their community. The research coordinator and each CBRA spoke every 1–2 weeks to discuss progress with the trial, and group teleconferences were held every 1–2 months to hear overall feedback from the CBRAs and address individual and group concerns or questions.

### Cervical cytology

In Ontario, Canada, to access the health system for Pap tests or other “medically necessary” procedures, all Ontario residents must have a health card that indicates they have Ontario Health Insurance and that they are eligible for Ontario health benefits (34–37). Some FN women can access cervical screening on-reserve, though most communities have limited healthcare resources on-reserve.

The sample collection methodology, interpretation of results, and follow-up communication in our study follows the current Ontario Cervical Screening protocol for primary care practitioners (31, 33). In our study, Pap tests are scheduled by CBRAs with local healthcare providers or participants’ preferred healthcare providers. Health care providers from neighboring communities will make visits upon requests of Health Directors to provide health care services to their FN communities who do not have these services locally. In these instances, the Research Team works with collaborating health care providers, CBRAs, and the CSC to coordinate community visits that would mirror the care that is normally offered on-reserve. In our study, Pap outcomes are communicated back to the health care providers, who are responsible for contacting individuals with abnormal results for follow-up, adhering to the Ontario Cervical Screening protocol for primary care practitioners (31, 33). Our focus group and interview discussions and consultations with the Aboriginal Health Initiative from the Society of Obstetricians and Gynaecologists of Canada (SOGC) emphasized the importance of accessible and culturally appropriate follow-up appointments. These discussions stressed that we need to help women understand the importance of attending a follow-up appointment to encourage attendance at these appointments. Consequently, we asked our collaborating healthcare provider to make follow-up appointments with the participants at the same time that the results are communicated.

### HPV self-sample testing

Participants pick up self-screening kits when they meet with the CBRA to complete the questionnaire and informed consent. The HPV sampling swabs are distributed to participants along with the collaboratively designed, instructional pamphlet (Zehbe et al. Manuscript in progress) for collecting a vaginal sample and a pre-postaged envelope to send off for testing. At the appointments, the CBRAs encourage participants to collect the sample while at the clinic (e.g., in a bathroom at the clinic) and to immediately mail their samples, though taking the kit home remains an alternative.

Swabs are tested at the British Columbia Center for Disease Control and positive high-risk HPV results from these tests are communicated with participants. Samples are then sent to the Dr. Alberto Severini’s laboratory at the National Microbiology Laboratory in Winnipeg, MN, Canada where genotyping of the samples are completed (38–44).

As suggested by the CSC, the Research Team has partnered with local healthcare providers who may provide medical care to women from our participating communities. On the ACCSS consent forms, participants are asked how they would like to have positive results communicated back to them, and the Research Team ensures that these wishes are met. Collaborating nurse practitioners and physicians work with the Research Team to communicate positive high-risk HPV results back to women and recommending them to attend a follow-up Pap test (11, 16, 34–36).

### Ethics approval

This project is conducted in accordance with the Tri-Council Policy Statement on Ethical Conduct for Research Involving Humans^1^. We have obtained ethics approval through the Lakehead University Research Ethics Board (#126 12-13/ROMEO #1463139) for the CRCT. The qualitative element of this project was jointly approved by Lakehead University Research Ethics Board and the Thunder Bay Regional Health Sciences Centre Research Ethics Board. Informed consent has been obtained from all participants of the qualitative and quantitative components of ACCSS. The trial registration number is International Standard Randomised Controlled Trial Number Register: ISRCTN84617261^2^.

## Results

Results are summarized in Table 2 below.

**Table 2:** **Summary of community engagement results in ACCSS**.

Component of ACCSS	Outstanding findings
Qualitative work	FN women experience social, environmental, and institutional barriers to cervical screening, including poor access to healthcare, legacy of colonialism, and impoverished living conditions
	Education before trial is crucial to help women understand why cervical screening is important
Integrating randomized trial design with participatory action approach	Frequent conversations with CBRAs and CSC introduced a tailored execution of the research project in each of the communities
Incentives	Using a lottery-based incentive program, prizes of increased value offer participants the opportunity to purchase groceries or other necessities that may not be available on-reserve
Questionnaire	Trial questionnaire focused on social and environmental factors related to cervical screening preferences
	Psychosocial section will gather information about participants’ experiences and anxieties with cervical screening
	Some participating women were forthcoming with personal histories of sexual abuse and violence, and wanted to include this information in the open-ended questions
	When administering the questionnaires, CBRAs ask participants if they want these personal experiences included in the questionnaire
Recruitment approach	Educational events facilitated by the CBRAs launched the trial in each community, helping raise awareness about cervical screening and build relationships between Research Team and community
Reflection meeting before Phase 2	Building upon a PAR approach, a meeting was held between the Research Team, CBRAs, and CSC to reflect on successes and improvements following the first offer of screening in communities
	“In-person” approaches were most successful at recruiting participants in Phase 1 and were part of the Phase 2 protocol
	There is a need for more community dialog between Research Team and community members and stakeholders. Discussing ongoing progress of ACCSS with communities will ensure that project is congruent with communities’ goals
Knowledge translation	At least three representatives from FN communities are invited to formally review publications and reports generated from ACCSS
	CBRAs and CSC will help tailor strategies for disseminating research findings
Participatory action approach	Community engagement is foundation of PAR and helps widen ethical space
	Social–ecological model depicts the multiple dimensions and targets of community engagement within ACCSS

### Qualitative findings: Knowledge, attitudes, and behaviors about HPV and cervical screening in our partner communities

Prior to the two-armed screening trial, a qualitative exploration was conducted between July 2011 and July 2012 by Ingeborg Zehbe and Marion Maar to shape the design and implementation of the trial [(20); Maar et al., under review; Wakewich et al., manuscript in progress; Zehbe et al., manuscript in progress]. With a tumor biologist whose expertise is cervical cancer and a medical anthropologist with expertise in Aboriginal research leading the field-work, this part of the study gained a particular inter-disciplinary rigor. The participants were eager to share their experiences with the team and welcomed the opportunity to ask medical questions about cervical cancer and HPV. Eighteen semi-structured interviews were held with community Health Directors and their designated key informants, which brought in a broad range of experiences from health care professionals, including social workers, physicians, nurse practitioners, community health nurses, health directors, and community health representatives. Nine community focus groups based on semi-structured guides were conducted on-reserve and involved women (3–10) from a variety of backgrounds, typically without a formal healthcare education.

After these meetings, community members, healthcare professionals, and the Research Team had the opportunity to reflect on our understanding of cervical screening in Ontario FN, and integrate these individualized needs into our CRCT component of this project. The social, environmental, and institutional barriers surrounding cervical screening that emerged from these community engagement efforts are described in detail elsewhere [(20, 30); Maar et al., under review; Zehbe et al., manuscript in progress]. From these discussions, it became overwhelmingly clear that creating awareness about cervical cancer and HPV through education was a priority across all communities. These findings culminated in a discussion group where community health directors and wellness workers and the Research Team actively reflected on the preliminary findings of the qualitative research and discussed the development of an educational strategy as a component of ACCSS. This collective effort transformed the trial methodology and incorporated a new health promotion element of our screening intervention, resulting in a culturally appropriate educational pamphlet designed by a local woman (Zehbe et al., manuscript in progress), complementary posters, as well as plans for community-specific educational events.

### Design and implementation findings

We have successfully launched a two-armed CRCT through the contributions of our FN community partners. Recruitment strategies and the offer of the cervical screening intervention have been tailored to the unique social and political environments in each community, shaping how ACCSS is implemented in each FN community. CBRAs and the CSC have identified the social and health disparities that their communities face with cervical cancer screening. In addition to the design, content, and presentation of our educational pamphlet (Zehbe et al., manuscript in progress), the CSC and CBRAs were instrumental in engaging community women to participate in ACCSS. Through transcript analyses, teleconferences, one-on-one discussions, and in-person group meetings, our community partners were actively involved in the determination of incentives, the execution of the recruitment strategies, and the planning of the second offer of cervical screening in the communities. The development and implementation of ACCSS is guided by the theory of *ethical space* (28, 29) in addition to the *socio-ecological* framework (Maar et al., under review).

### Incentives

Integrated efforts with the Research Team and CSC led to the construction of the ACCSS protocol for the randomized trial. Promotion of the educational event and study invitations, data collection processes, and disseminating research results were discussed and agreed upon by the CSC and Research Team. This approach also ensured that the research process was transparent and understandable by lay individuals. The CSC relayed that incentives are essential for engaging women from our partner FN communities to participate in ACCSS. We jointly decided on a lottery incentive system such that we would award a $50 gift card for a large discount department store to 1 person for every 10 consent forms we received from each community. The lottery method allowed the Research Team to give out larger incentives and the CSC felt that the store selection would encourage winners to purchase groceries and supplies that are not easily accessible on-reserve. To encourage participation in follow-up, a grand prize draw will be held after the final data collection point for all participants who completed all three questionnaires (i.e., baseline questionnaire plus two rounds of follow-up).

### Questionnaire

The questionnaire for the randomized controlled trial was designed by the Research Team and the CSC, with final review conducted by the CBRAs. Heavily informed by the qualitative results, the baseline questionnaire aims to collect data on the following domains: social-demographic information, personal health behavior, feelings toward women’s healthcare, psychosocial health, and knowledge of HPV and cervical cancer. The influence of social circumstances on women’s health choices became abundantly clear during the qualitative component of ACCSS, and as a result, we aimed for the questionnaire to collect detailed information about the impact of cultural, social, and political environments on their cervical screening choices. Follow-up questionnaires are tailored to each community in terms of study timeframe and method of cervical screening first offered. We will repeat measurements of psychosocial health and gather information about the participants’ experiences of cervical screening from the trial through these follow-up questionnaires. Similar to the baseline questionnaire, many questions are open-ended to provide flexibility to truly capture the depth of ACCSS participants’ experiences. Please see Data Sheet S1 in Supplementary Material for the baseline questionnaire, and a follow-up questionnaire for a community first offered HPV self-sampling.

Most notably, after the completion of the first round of cervical screening offers, the CBRAs have indicated that some participants are questioning the lack of questions surrounding sexual abuse, substance abuse, and violence in the ACCSS questionnaire, during the interview between the CBRA and the participant. Because this topic is highly sensitive and stigmatized, we have discussed strategies with the CBRAs and the CSC for capturing this information that some participants express as a major influence in their cervical screening preferences, without offending other participants. Consequently, we have several open-ended questions that we have guided our CBRAs to fill in with these additional supplied details *only if the participant explicitly agrees to include their story*. To avoid exploiting the personal and trusting space built between the CBRA and the participant, we and our community partners agreed that the participant should be asked if they wish to include their experiences in the questionnaire. On the other hand, some participants agreed to complete the questionnaire under the condition that they could fill it out without the CBRA seeing the responses. Because the CBRAs were known within the community, this offered some benefits, but also some drawbacks as some potential participants worried about their confidentiality.

### Recruitment approach

At the training, CBRAs started to develop their proposals for educational events that would launch the trial in their community. Once the CBRAs had received training about HPV, cervical cancer, and the study protocol, we shared the community randomization result with the Chiefs, Councils, Health Directors, and the CBRAs. Potential participants were not informed that the reverse cervical screening method would be offered in their community in the second screening phase to prevent preference bias.

With these outlines, CBRAs indicated the resources they needed from the Research Team and from their community organizations and institutions, and how they would execute these educational events in the following months. The CBRAs created community educational events that reflected their local conditions and programing (see Data Sheet S2 in Supplementary Material for a description of the invitational events in our partner communities). Members of the CSC (e.g., Health Directors and healthcare providers) and political leaders also helped to integrate these events into the already established community health programing. These events were promoted in the community by the CBRA using a variety of methods, including social media, posters around the community and band offices, door-to-door visits, phone calls, attendance at community celebrations and Pow-Wows, and newsletter inserts.

Beginning in May 2013, our 11 partner communities started the trial locally with these community educational events, led by the CBRAs. Though each event was unique to the local community and environment, common to all sessions were:

- An introduction of the CBRA as a representative of the local health team and the Research, and an introduction of Research Team members who attended (Brianne Wood and Ingeborg Zehbe)
- A presentation about HPV, cervical cancer, available cervical screening options, and how individuals can participate in the study using a life-size model of the female genital tract for demonstration purposes
- A question and answer period for study-related or health-related questions
- A complimentary meal or refreshments, often prepared by someone in the community
- Additional ACCSS resources, including our ACCSS pamphlets, study t-shirts featuring our logo painted by a local artist with copyright obtained exclusively by the ACCSS Research Team, and information sheets
- An invitation for eligible women to contact their CBRA about participating in cervical screening.

At the end of the events, women who were interested in learning more about the study provided their names and contact information to the CBRA, who would follow-up with them to make appointments to complete the consent forms and questionnaires.

Community-Based Research Assistants would ensure that the women were eligible when scheduling individual appointments with these women after the event, and would bring a study package to their meeting. The study packages included an informed consent form, a questionnaire, and an HPV self-sampling kit with a pre-addressed, postage-paid envelope for the HPV-test assigned communities. The CBRAs also brought study information pamphlets and a small model of the female reproductive system to meetings so that they could try to answer questions during the meeting, or would have the contact information of the Research Team if any difficult questions arose. The informed consent process was explained thoroughly, and the baseline epidemiologic questionnaire was administered by the CBRA to consenting women. Women were then offered to take part in the cervical screening modality that their community was randomly assigned through the trial. In HPV-randomized communities, participants were encouraged to take their samples during their meetings with the CBRA, and packages were either handed back to the CBRA to mail or the participants mailed the samples themselves. In Pap test communities, the CBRAs offered participants help with scheduling an appointment with their personal healthcare provider or one of the project’s collaborating health care providers.

The CBRAs continued to reach out to eligible women in their community for 3 months following their initial educational event, using some of the same methods described above and working in congruence with the programs run by the community Health Directors. All of the promotional and recruitment efforts are recorded in the CBRA logbooks, which are submitted to the Research Team at the end of each phase.

### Reflection meeting preceding phase 2 of screening

The CBRAs, members of the CSC, and the Research Team met in September 2013 to reflect on the progress of the first phase and to inform the implementation strategy for the second phase. By identifying the site-specific cervical screening participation barriers, we decided to intensify the recruitment offer in Phase 2 of ACCSS. We heard that “in-person” communication was the most effective way to reach FN women (Maar et al., under review; Zehbe et al., manuscript in progress), which was subsequently incorporated into our Phase 2 protocol. At the reflection meeting CBRAs decided schedules to invite non-responders to participate through door-to-door communication, personalized phone calls with help from the band offices, and frequent newsletter messages. This more aggressive recruitment approach will standardize recruitment strategies between communities, and the in-person approach represents the “ideal” conditions for inviting women to participate in cervical screening. These attempts to reach more women with the offer of cervical screening are important to our partner communities and offer methods of health promotion in FN communities.

Our community partners also emphasized the importance of disseminating our current findings with community stakeholders and members prior to offering the second round of screening. Communicating the progress, findings, and future directions of ACCSS was stressed during the meeting as another method of engaging women from the community. Re-establishing ties with the political leaders and other stakeholders in the community was described as important for laying the foundation for future partnerships with the communities but also to ensure that the priorities of the project and the communities are congruent.

### Knowledge translation

Flexibility and open dialog are not only important to the internal study structure, but also to encourage communication between external policy-makers. With the support of community political leaders and healthcare professionals, the strategic collaborations with key stakeholders such as the SOGC and the Assembly of FN (AFN) will ensure a fast translation of ACCSS results into FN Health policy and practice so that this study can inform a larger scale application in other FN communities. At least three women from our partners and community members are selected to participate in the Publication Steering Committee (PSC) to critique each publication or report. The PSC members are offered the position of an author. PSC members will help plan dissemination at scientific meetings and conferences; notes in special-interest newsletters or magazines; articles and features. For community/regional dissemination, the CBRAs and CSC provide insight about a schedule of dissemination events that fit the needs and situations of their respective communities. Presentations will also be offered at community meetings, regional Chiefs’ Meetings, and/or FN health planning meetings. To ensure that results will influence policy, programs, and practice, a multi-pronged policy impact dissemination strategy will be designed with the aforementioned key stakeholders, the CSC, and respective Chiefs and Councils. These strategies will include educational sessions with the local medical school as well as nation dissemination with women’s healthcare professionals. Additionally, we will ask our community partners to translate their learned knowledge back to the Research Team, to inform future research processes.

Maintaining an open dialog with community partners and stakeholders has been important for widening the *ethical space* of the project, and ensuring a rich exchange of knowledge between community partners and the Research Team (28, 29). With the foundation of the project rooted in ethical space, the successful engagement of the CSC, CBRAs, and additional community stakeholders has allowed for a smooth amalgamation of qualitative and quantitative research in ACCSS. Following the final offer of screening, *triangulation* will be conducted between the qualitative and quantitative outcomes to explore how *knowledge, attitudes*, and *behaviors* surrounding cervical cancer screening and HPV in our partner communities impact the health status of FN women and communities. The mixed-methods approach compliments the rigours of the randomized controlled trial by acknowledging the dynamic and social nature of health programing.

In ACCSS, we also observed that the integrated contributions of the CBRAs, the CSC, and political leaders including Chiefs and Councils fit the theoretical framework of the socio-ecological model (Maar et al., under review). These three distinct community-based representations offer distinct and significant perspectives that are critical for transforming the cancer screening culture and reducing the burden of HPV and cervical cancer in our partner communities. The CBRAs represent the innermost circles of the socio-ecological model, reflecting the individual and family knowledge, attitudes, and beliefs toward cervical cancer screening. The CBRAs are committed to reaching out to women and families, recruiting women to participate in cervical screening and promoting health behaviors at the individual level. It then follows that the CSC – composed of healthcare providers and community health representatives – should be focused on capacity building at the organizational level. The members of the CSC are committed to improving the health of their community and have the knowledge, experience, and drive to execute ACCSS at the community level. Additionally, they are careful and protective when communicating with the Research Team, and demand transparency and compromises between scientific rigor and the implementation of public health programing. At the outermost level, the political leaders of the FN communities reflect individuals who can advocate for policy and structural change. The Chiefs and Councils of our partner communities want assurance that the direction and overall objectives of the project remain clear – to improve the health and overall welfare of FN people. With the insight and support from these stakeholders, as well as the other community partners, ACCSS can effectively advocate for improved healthcare at the policy level.

## Discussion

Anishinaabek Cervical Cancer Screening Study is the first community-randomized trial in Canada under a PAR framework to explore how knowledge, attitudes, and behaviors of FN women influence cervical screening preferences. We continued to build relationships beyond the pilot study and the formal execution of the study (28, 30) throughout the qualitative component of the project. The need to build awareness on cervical cancer through educational events stood out from the interviews and focus groups, and was consequently integrated into the design of the two-armed CRCT.

By acknowledging local conditions and unique healthcare systems, the PAR process provides us with a deeper understanding of the factors that are important for improving cervical screening uptake in FN women. ACCSS can provide generalizable evidence through its mixed-methods design that can inform cervical screening guidelines and policy for high-risk populations, such as Canadian Aboriginal women, by considering the complexities that are inherent to their surrounding environment (45, 46). Building upon the ethical space framework and the socio-ecological model [(28); Maar et al., under review], expanding the community networks to include advocates for individuals and families, community organizations, and policy change created a dynamic cervical screening intervention that has a higher probability of reaching the underscreened population than a traditional scientific intervention study. Engaging in equitable and flexible relationships has led to transparent research processes and it built a foundation of trust that has been established between the Research Team and partner FN communities. With histories of disenfranchisement, shared decision-making ensures that the FN communities can maintain the integrity of their culture while the scientific integrity of ACCSS is also maintained.

Involving community-based representatives to implement the trial within their community is important for engaging individual women and the community in cervical screening and the research project, though there are considerable challenges and limitations to the integrated community engagement and controlled trial approach (27). Scheduling and coordinating multiple networks of community partners in addition to a Canada-wide research team means that making decisions and implementing changes take considerably longer than a traditional CRCT. Also, due to resulting variability in the recruitment strategies and individual efforts of the CBRAs, we expect a differential effect of recruitment within the different communities. The community randomization design should accommodate for this variability as we expect there to be similar variability in CBRA efforts in each arm. However, with our integrated PAR and CRCT approach, this flexibility may be more effective for informing a long term solution by demonstrating effective approaches at health system changes by focusing on relationships and communication.

The integrated participatory and CRCT design also invited potential threats to internal validity if there were deviations from the protocol. However, CBRAs were usually well-known in their community and they were usually able to create a trusting environment in which they reach potential participants, and provide support for their local health program. These personalized and intensive recruitment efforts of the CBRAs within the communities respect the dynamic nature of healthcare systems and allow changes to be made from inside the communities. These “soft” outcomes require flexibility on the academic side when interpreting ACCSS results and measurements of “success.”

Because socio-economic, historical, and cultural variables have a well-established link to health outcomes and quality of life (18, 19, 21), we can learn from the ways they apply cervical screening interventions in their unique social environments. Being flexible in a research approach and sharing decision-making across the many levels of community participation is important for understanding the inequities that FN peoples face and empowering these communities to overcome cervical cancer disparities. Respecting the diversity that each FN community represents and the contexts in which they live will help academics, community leaders and policy-makers offer improved cervical cancer screening and future and superior healthcare in general.

## Author Contributions

The project was initially conceived by Ingeborg Zehbe. These authors (in alphabetical order) all have contributed to the design and implementation of the ACCSS study: Ann N Burchell, Julian Little, Marion Maar, Gina Ogilivie, Alberto Severini and Ingeborg Zehbe. Nicholas Escott has contributed to the implementation of ACCSS and has a role as clinical advisor in ACCSS. These authors (in alphabetical order) have contributed to the writing of the manuscript: Ann N Burchell, Brianne Wood and Ingeborg Zehbe. Kyla Morrisseau and Lisa Bishop were part of the Publication Steering Committee and helped shape the manuscript and member-checked reported details. All authors read and approved the final manuscript.

## Conflict of Interest Statement

The authors declare that the research was conducted in the absence of any commercial or financial relationships that could be construed as a potential conflict of interest.

## Supplementary Material

The Supplementary Material for this article can be found online at http://www.frontiersin.org/Journal/10.3389/fonc.2014.00027/abstract

## Footnotes

^1^http://www.ethics.gc.ca/pdf/eng/tcps2/TCPS_2_FINAL_Web.pdf

^2^http://isrctn.org/
