# Supplementary material for: Using Community Engagement to Inform and Implement a Community-Randomized Controlled Trial in the Anishinaabek Cervical Cancer Screening Study
Source: Front Oncol. 2014 Feb 19;4:27. doi: 10.3389/fonc.2014.00027 (PMC3928568; doi:10.3389/fonc.2014.00027)
Supplement: Supplementary file 1 [file 74074_Zehbe_DataSheet1.PDF]

Thunder Bay Regional Research Institute

Anishinaabek Cervical Cancer Screening Study Questionnaire

Delivered by (name and community):

Date and time started: Date and time completed:

Thank you very much for agreeing to complete this survey for the Anishinaabek Cervical Cancer Screening Study. Your help will ensure that the study will be able to answer questions about how cervical cancer screening fits into your community and the Aboriginal holistic health perspective.

The survey will ask questions about you, your health and health practices, your knowledge of and attitudes toward human papillomavirus (HPV), cervical cancer screening and women's health in general. It should take about 30 minutes to complete. Please let me know if you don't feel comfortable answering any of the questions – you do not need to answer questions that make you uncomfortable. Most questions require that you simply indicate which answer fits best with your experience. Other questions ask for you to select all of the options that apply to you. There are also a few questions where you may be asked to talk about any experiences that were not covered by a particular question or section. Depending on your answer for some questions, I may skip past some questions or go to a different part of the questionnaire.

There are no right or wrong answers to any question. Since we will be using this survey with many women with different experiences, you may find that some of the questions do not seem to apply to you. Other questions will definitely be relevant. Many questions ask you to think back over your adult years, or over the past several months, to recall specific information. Please take your time to consider each question carefully.

Remember that all your answers are completely confidential. We encourage you to try to answer all questions but you do not need to answer questions that make you feel uncomfortable or you do not know. A good guess is always better than no information at all. If you would like to tell us more about any specific items, please use the available space at the end of the questionnaire. If you have questions during the questionnaire, please feel free to ask the interviewer or you may contact the research team directly or using the help-line as indicated on the consent form.

Let's begin!

Thunder Bay Regional
Research Institute

Personal Health

I'm going to ask you some questions are about your medical and health history as well as your access to health resources.

1. In general, compared to other people of your age, would you say your health is:
    a. Excellent
    b. Very good
    c. Good
    d. Fair
    e. Poor

2. In general, what best describes your health care situation (select all that apply):
    |_|I visit the health care centre in the community
    |_|I use the services off reserve using the community's transportation services
    |_| I use the services off reserve but use my own transportation
    |_| I access traditional medicinal practices or traditional healing in my community
    |_| I access traditional medicinal practices or traditional healing outside of my community

3. A Pap test is a test where a doctor or nurse practitioner checks for changes in the cells of the cervix. The cervix is the lower part of the uterus/womb that opens into the vagina, the birth canal. A Pap test can be done during a pelvic exam or a Well-Woman exam. (Please provide pamphlet as visual aid)
    Have you ever had a Pap test:
    a. Yes
    b. No (skip to #7)
    c. Do not know (skip to #7)

4. When was the last time you had a Pap test:
    a. Less than six months ago
    b. Between six month and 1 year ago
    c. 1 to 3 years ago
    d. More than 3 years ago
    e. Do not know

5. Have you ever been told that you need to have follow-up to a Pap test or told that you have had an abnormal Pap test:
    a. Yes
    b. No (skip to #7)
    c. Do not know (skip to #7)
    d. Prefer not to answer (skip to #7)

6. If you have had an abnormal Pap test, what best describes your present situation:
    a. I am waiting for or I am still receiving treatment from my doctor
    b. I have not followed up these tests results because I have not been able to get services
    c. I decided not to have treatment because I am uncomfortable with it
    d. I decided not to have treatment because I don't think it will help
    e. I have completed treatment
    f. Other (please specify) ----------------------------→_________________________
    g. Do not know
    h. Prefer not to answer

One method of cervical screening being used in this study is a self-sampling test that allows women to take a sample of cells from their vagina to be tested for the virus that causes cervical cancer. This test requires women to insert a swab into their vagina and rotate a few times, and then closing this swab in a test tube to send off to a lab. Using this self-sampling test can be compared to using a tampon during a menstrual period or "Moon-Time". (Please provide pamphlet as visual aid)

7. Have you ever used tampons:
    a. Yes
    b. No
    c. Prefer not to answer

The cervix is the connection between the uterus, where the baby grows inside a woman, and the vagina is the canal where a baby is born. Some women have a surgery called a hysterectomy to remove the uterus for medical reasons or by choice. Different methods of removing the uterus affect women's risk of getting cervical cancer. (Please use pamphlet as visual aid of anatomy)

8. Have you had a hysterectomy:
    a. Yes
    b. No
    c. Do not know
    d. Prefer not to answer

Participant ID:
Date started: **Psychosocial Health**

Feelings toward Women's Health Care

The following questions are asking about *your* feelings and experiences with health care related to women's health issues. If you have had a Pap test before or a Well Woman visit, try to recall your experience with those health visits. Please indicate how much you agree with the following statements by checking only one box per row. The accompanying scale may help you visualize your answer. If you feel that a question doesn't apply to you, please let me know.

	**Strongly Disagree 1**	**Disagree 2**	**Slightly Disagree 3**	**Neither agree nor disagree 4**	**Slightly Agree 5**	**Agree 6**	**Strongly Agree 7**	**Prefer not to answer**
I feel comfortable discussing women's health issues with a male health professional								
I think I have a trusting relationship with my healthcare provider								
I often feel that the health services that I receive are respectful of my culture and heritage								
Scheduling medical appointments is difficult because I find it hard to get childcare								
Scheduling medical appointments is difficult because I find it hard to take time away from my job								

Participant ID:
Date started:

Psychosocial Health

	**Strongly Disagree** 1	**Disagree** 2	**Slightly Disagree** 3	**Neither agree nor disagree** 4	**Slightly Agree** 5	**Agree** 6	**Strongly Agree** 7	**Prefer not to answer**
I cannot access the health services in my community								
Growing up, my mother/caregiver did not discuss personal women's issues such as sexuality with me								
I am afraid that my visit or results will not be kept private								
I am able to/I hope to be able to be open to discussing personal women's issues with my children (this may not be applicable for you – please indicate)								
I do not have access to transportation for out-of-town medical appointments								
I know why Pap tests are important								
I believe Pap tests are important for me								

Participant ID:
Date started:

Psychosocial Health

	**Strongly Disagree 1**	**Disagree 2**	**Slightly Disagree 3**	**Neither agree nor disagree 4**	**Slightly Agree 5**	**Agree 6**	**Strongly Agree 7**	**Prefer not to answer**
I think it is important to look after my health so I can be a role model for my children and/or young people in the community								
I may or may not get cancer, but there isn't much I can do about it								
If I had cancer, I would trust the doctors would do their best to cure me								
I think that traditional Anishinaabek medicine is important for staying healthy and preventing disease								

Participant ID:
Date started:

Psychosocial Health

This section of the questionnaire asks about how you feel about your health and your body. How you think and feel are an important part of overall health and wellness. Please indicate how much you agree with the following statements (the attached scale can be used as a visual aid).

	**Strongly Disagree 1**	**Disagree 2**	**Slightly Disagree 3**	**Neither agree nor disagree 4**	**Slightly Agree 5**	**Agree 6**	**Strongly Agree 7**	**Prefer not to answer**
In the last three months, I have been worried about my general health								
In general, I feel well informed about cervical screening								
I think that I will probably get some form of cancer in my life								
In the last three months, I feel good about my body								
In the last three months, I have worried about getting a positive cervical screening test result								
In the last three months, I have worried about my ability to have children								

Participant ID:
Date started:

Psychosocial Health

	**Strongly Disagree**<br><br>**1**	**Disagree**<br><br>**2**	**Slightly Disagree**<br><br>**3**	**Neither agree nor disagree**<br>**4**	**Slightly Agree**<br><br>**5**	**Agree**<br><br>**6**	**Strongly Agree**<br><br>**7**	**Prefer not to answer**
I think that my chances of getting cervical cancer are much higher compared to other women								
In the last three months, I am generally satisfied with the support I have had from other people								
In the last three months, I have worried about my family or other people in my community learning about my cervical screening results								

HPV and Cervical Cancer Information

1. How did you learn about HPV or cervical cancer (select all that apply):
   |_| My health care provider
   |_| Friend or family member
   |_| Sexual education in school or workshop run by the health clinic
   |_| Internet (website or social media)
   |_| TV
   |_| Poster or pamphlet
   |_| I don't really know much about HPV or cervical cancer
   |_| Other (please specify) ---------------------------------→ ______________________
   |_| Prefer not to answer

We are now going to ask you a few questions about HPV, cervical cancer and cancer screening. We will provide a fact sheet with the correct answers afterwards. Please try to answer as many questions with a "true" or "false" as you can. If you have any additional questions, please feel free to contact someone on the research team or www.accssfn.com

	**True**	**False**	**Not sure**
Most people will get HPV at some point in their life.			
People get HPV by sharing a fork, plate or glass with someone who has HPV.			
HPV infections are the main cause of cervical cancer.			
HPV only affects women.			
Pap tests and HPV tests help prevent cervical cancer.			

Social and Demographic Information

These questions are about where you live and your background. These questions will help us understand a bit more about the people who are participating in the study.

1. What is your age group:
    a. 25-29
    b. 30-34
    c. 35-39
    d. 40-44
    e. 45-49
    f. 50-54
    g. 55-59
    h. 60-64
    i. 65-69

2. What First Nations community/communities are you registered with:
____

3. Do you currently live on-reserve:
    a. Yes
    b. No, I live: --------------------------→ ___________________________(where you live now)

4. Do you consider yourself:
    a. First Nation
    b. Métis
    c. Do not know
    d. Prefer not to answer
    e. Other (please specify) --------------------------→ ___________________________

5. In what language are you are most comfortable speaking:
    a. English
    b. Ojibway, or another Anishinaabek language
    c. French
    d. Other (please specify) ----------------------------> _____________________________

6. What is the highest level of education that you have completed:
    a. Less than high school
    b. High school diploma
    c. Some trade, technical school or college
    d. Received certificate from university or diploma from college
    e. University degree

7. Do you currently work for pay (e.g., wages, salary, self-employed):
    a. I am currently employed
    b. I am not currently employed, and I am looking for paid work
    c. I am not currently employed and I am *not* looking for paid work (Note: this includes students, retirees, homemaker, etc)
    d. Prefer not to answer

8. How many children live in this household? *Include all children, 17 or younger, who reside in this household at least half of the time. If none, mark "0":*

0-5 years	
6-11 years	
12-17 years	

9. Including you, how many adults live in this household? *Include all adults, 18 and over, who reside in this household at least half of the time:* ______

In the interviews and focus groups, we heard that attending a residential school has had a lasting impact on the Aboriginal culture, First Nations women in particular. The term "residential school" refers to residential school systems attended by Aboriginal students. This includes residential schools run by religious orders, industrial schools, boarding schools, student residences, hostels and billets. The last residential school shut down in 1996. ***Remember that your answers are confidential.***

10. Have you ever attended a residential school?
    a. Yes
    b. No
    c. Do not know
    d. Prefer not to answer

11. Have your parents or grandparents ever attended a residential school?
    a. Yes
    b. No (skip to next section)
    c. Do not know (skip to next section)
    d. Prefer not to answer (skip to next section)

12. Do you think that your overall health and well-being has been affected by personally attending or having a family member attend a residential school?
    a. Yes, negatively impacted
    b. Yes, positively impacted
    c. No impact
    d. Do not know
    e. Prefer not to answer

13. Do you have any additional comments about any question you have seen so far on this questionnaire:
    ____
    ____

Food Security

Food security has been defined as: "…. when all people, at all times, have physical and economic access to sufficient, safe and nutritious food to meet their dietary needs and food preferences for an active and healthy life" (World Food Summit, 1996). Food security is related to good health.

This last section asks questions about being able to afford food for your household. Some of the questions are very personal and may be difficult for you to answer. Like the rest of the questionnaire, this information is strictly confidential and no names will be released to the community or government.

I'm going to read several statements that may be used to describe the food situation of a household. The following questions ask about your access to food over the past 12 months.

1. Which of the following statements best describes the food eaten in your household in the past 12 months?
a. You and other household members always had enough of the kinds of food you wanted to eat.
b. You and other household members had enough to eat, but not always the kinds of food you wanted.
c. Sometimes you and other household members did not have enough to eat.
d. Often you and other household members didn't have enough to eat.
e. Do not know
f. Prefer not to answer

The following statements may be used to describe the food situation for a household. Please indicate if the statement was often true, sometimes true, or never true for you and other household members in the past 12 months.

2. You and other household members worried that food would run out before you got money to buy more. Was that often true, sometimes true, or never true in the past 12 months?
a. Often true
b. Sometimes true
c. Never true
d. Do not know
e. Prefer not to answer

3. The food that you and other household members bought just didn't last, and there wasn't any money to get more. Was that often true, sometimes true, or never true in the past 12 months?
a. Often true
b. Sometimes true
c. Never true
d. Do not know
e. Prefer not to answer

4. You and other household members couldn't afford to eat balanced meals. In the past 12 months was that often true, sometimes true, or never true?
a. Often true
b. Sometimes true
c. Never true
d. Do not know
e. Prefer not to answer

If the participant responds "often true" or "sometimes true" to ANY ONE of #1-4,

OR "Sometimes" or "Often" to #1, then continue to #5; otherwise, skip to the next section.

The following questions are about the food situation in the past 12 months for you or any other adults in your household.

5. In the past 12 months, did you or other adults in your household ever cut the size of your meals or skip meals because there wasn't enough money for food?
a. Yes
b. No (Skip to 9)
c. Do not know
d. Prefer not to answer

6. How often did this happen?
a. Almost every month
b. Some months but not every month
c. Only 1 or 2 months
d. Do not know
e. Prefer not to answer

7. In the past 12 months, did you personally ever eat less than you felt you should have because there wasn't enough money to buy food?
a. Yes
b. No
c. Do not know
d. Prefer not to answer

8. In the past 12 months, did you personally lose weight because you didn't have enough money for food?
a. Yes
b. No
c. Do not know
d. Prefer not to answer

If the participant responded “yes” to #5, #7 or #8, continue to #9; otherwise, skip to the next section

9. In the past 12 months, did you or other adults in your household ever not eat for a whole day because there wasn’t enough money for food?

a. Yes
b. No
c. Do not know
d. Prefer not to answer

10. How often did this happen?

a. Almost every month
b. Some months but not every month
c. Only 1 or 2 months
d. Do not know
e. Prefer not to answer

Questionnaire Evaluation

To help us make better questionnaires in the future, we would like to hear your feedback on the current questionnaire.

	**Strongly Disagree**<br><br>**1**	**Disagree**<br><br>**2**	**Slightly Disagree**<br><br>**3**	**Neither agree nor disagree**<br>**4**	**Slightly Agree**<br><br>**5**	**Agree**<br><br>**6**	**Strongly Agree**<br><br>**7**
The questions that were asked in this questionnaire were relevant to my health and to cervical cancer screening.							
The questions in this questionnaire pertaining to my health were straightforward and easy-to-answer.							

1. Do you have any other comments about the questionnaire or your experience in the study (please specify):

____

____

Miigwetch. Thank you for your participation with the study.

Participant ID:
Date started: **Follow-up Questionnaire #1**

Follow-up Survey #1 about experience in cervical cancer screening

Thank you for participating in follow-up in the Anishinaabek Cervical Cancer Screening Study. We would like to hear about your experience as a participant with HPV self-sampling from May 2013 to August 2013.

1. Have you attended a Pap test between May 2013 and August 2013?
   a. Yes
   b. No (skip to Question 3)
   c. Do not know (skip to Question 3)
   d. Prefer not to answer (skip to Question 3)

2. Did you have the Pap test done because (please select all that apply):
   a. The Pap test was offered as part of the Anishinaabek Cervical Cancer Screening Study
   b. You were due to have a Pap test done
   c. Other (please specify) ----------------------------→__________________________
   d. Do not know

3. Did you choose to participate in the self-sampling screening offer in your community between May 2013 and August 2013?
   a. Yes
   b. No
   c. Prefer not to answer

If you chose to participate in self-sampling, why? If you chose not to participate in self-sampling, why not?

____

____

4. If you answered "Yes" to Question 3: When participating in the screening, how did you feel during your self-sampling experience?
   a. Very comfortable
   b. Comfortable
   c. Neither comfortable nor uncomfortable
   d. Somewhat uncomfortable
   e. Very uncomfortable

f. Prefer not to answer

5. Why did you feel this way with after using the self-sampling test?

____

____

Some communities in our study have been offered the self-sampling test as a method of cervical screening. This test looks for the high-risk types of human papillomavirus (HPV). This method can be done at home or in a clinic, without a doctor. (Please see visual aid)

6. If you have the option of using self-sampling as a tool for screening for cervical cancer, how would this affect you in the future?
    a. I would use the self-sampling tool for cervical cancer screening **in addition to** other required visits to my doctor (e.g., Well-Woman exams)
    b. I would use the self-sampling tool for cervical cancer **instead of** my current visits to the doctor
    c. I would prefer to continue visiting my health care provider for all of my health issues.
    d. Do not know

7. Would a reminder make you more likely to participate in cervical cancer screening?
    a. Yes
    b. No
    c. Not sure

8. In the future, would you be more likely to participate in cervical cancer screening if you were mailed a reminder letter, sent a reminder email, or had a health care provider phone or text you?
    a. I would prefer a mailed reminder letter.
    b. I would prefer a reminder email.
    c. I would prefer a reminder telephone call
    d. I would prefer a reminder text.
    e. Other (please specify) ---------------------------→__________________________
    f. Do not know

9. Who would you be comfortable with contacting you with a reminder for cervical cancer screening (please check all that apply):
    a. My health care provider (doctor or nurse practitioner) at an off-reserve clinic

b. A health clerk at an off-reserve clinic
c. A visiting health care provider(doctor or nurse practitioner) in your community
d. Your community health nurse
e. Your community health representative
f. A health clerk in your community

10. What are some important reasons for you deciding to participate in the study (please check all that apply):
    a. My health is important to me
    b. I want to help improve the health in my community, of my children, of the next generation
    c. I think cancer screening is important to help bring health and healing back into the community
    d. My friends or family wanted to me to join the study
    e. I was interested in winning the incentive
    f. Other (please specify) ----------------------------→__________________________
    g. Do not know
    h. Prefer not to answer

Participant ID:
Date started: **Follow-up Questionnaire #1**

Psychosocial Health

This section of the questionnaire asks about how you feel about your health and your body. How you think and feel are a very important part of overall health and wellness. Please indicate how much you agree with the following statements (the attached scale can be used as a visual aid).

	**Strongly Disagree** 1	**Disagree** 2	**Slightly Disagree** 3	**Neither agree nor disagree** 4	**Slightly Agree** 5	**Agree** 6	**Strongly Agree** 7	**Prefer not to answer**
In the last three months, I have been worried about my general health.								
In general, I feel well informed about cervical screening.								
I think that I will probably get some form of cancer in my life.								
In the last three months, I feel good about my body.								
In the last three months, I have worried about getting a positive cervical screening test result.								
In the last three months, I have worried about my ability to have children.								

Participant ID:
Date started: **Follow-up Questionnaire #1**

	**Strongly Disagree 1**	**Disagree 2**	**Slightly Disagree 3**	**Neither agree nor disagree 4**	**Slightly Agree 5**	**Agree 6**	**Strongly Agree 7**	**Prefer not to answer**
I think that my chances of getting cervical cancer are much higher compared to other women.								
In the last three months, I am generally satisfied with the support I have had from other people.								
In the last three months, I have worried about my family or other people in my community learning about my cervical screening results.								

1. Is there anything else you wish to comment on today regarding your experience with women's health?

____

____

Miigwetch. Thank you for your participation with the study.
