# Supplementary material for: Using Community Engagement to Inform and Implement a Community-Randomized Controlled Trial in the Anishinaabek Cervical Cancer Screening Study
Source: Front Oncol. 2014 Feb 19;4:27. doi: 10.3389/fonc.2014.00027 (PMC3928568; doi:10.3389/fonc.2014.00027)
Supplement: Supplementary file 2 [file 74074_Zehbe_DataSheet2.PDF]

Supplementary Material. Table of Educational Events in Each Partner Community.

Host First Nations Community	Date of Educational Event	Description of Educational Event	Location of event
Animbiigo Zaagi'ing Anishinaabek (Lake Nipigon)<br><br>Biinjitiwabik Zaaging Anishinaabek (Rocky Bay)<br><br>Bingwi Neyaashi Anishinaabek (Sand Point)	**May 17, 2013**	**A celebration of women's health**<br><br>- Party atmosphere: decorations, party games, door prizes<br><br>- Traditional meal served (provided by host CBRA and family<br><br>- Well attended by host reserve: approximately 35 women	On-reserve in Biinjitiwabik Zaaging Anishinaabek<br><br>(transportation was required for Animbiigo Zaagi'ing Anishinaabek and Bingwi Neyaashi Anishinaabek members)
Fort William First Nation	**May 8, 2013**	**Information session and BINGO game**<br><br>- Light refreshments provided<br><br>- Original BINGO game with cervical cancer vocabulary created by host CBRA<br><br>- Many healthcare representatives attending, only 1 potential participant	On-reserve at Community Centre
Kiashke Zaaging Anishinaabek (Gull Bay)	**June 13, 2013**	**Lunch and Learn session**<br><br>- focus group atmoshphere: general discussion following slideshow presentation by the Research Team	On-reserve at Community Centre

		- Traditional meal provided by local cook<br><br>- Many of the attendees had been recruited through door-to-door invitations earlier in the day, though posters were put up in the community	
Long Lake #58 First Nation	**May 8, 2013**	**Lunch and Learn**<br><br>- focus group atmosphere: attendees were engaged in discussion about cervical cancer; Host CBRA presented cervical screening poster; Research Team answered questions<br><br>- Lunch arranged by host CBRA and family<br><br>- Well attended. Approximately 35 women attended the presentation, and many women brought their children and infants so that they could attend the meeting	On-reserve at Community Centre
Pays Plat First Nation	**May 22, 2013**	**Dinner Event**<br><br>- focus group atmosphere: women openly asked questions related to cervical screening following slideshow presentation<br><br>- Meal prepared by host CBRA	On-reserve at community centre

		- Well attended. Almost all eligible women in the community attended.	
Pic Mobert First Nation	**July 24, 2013**	**Lunch and Learn**<br><br>- Presentation oriented: Research Team gave slideshow presentation, and a few questions about cervical screening were asked following the presentation.<br><br>- Lunch was provided by the host CBRA<br><br>- Most of the attendees were employees or students from the Health Centre.	On-reserve in Health Centre
Ojibways of Pic River	**June 2, 2013**	**Afternoon Presentation**<br><br>- focus group/party atmosphere: Research Team gave a presentation, and a discussion about cervical cancer followed. There were door prizes and games, arranged by the host CBRA.<br><br>- Light refreshments were brought to presentation<br><br>- Another community event was taking place this day which may have affected the number of attendees. Approximately 10 attendees.	On-reserve at Community Centre
Red Rock First Nation (Lake Helen)	**May 22, 2013**	**A celebration of women's health**<br><br>- Party atmosphere: decorations, door	On-reserve at Community Centre

		prizes, musical performances<br><br>- Traditional dinner provided by host CBRA and family<br><br>- A cervical cancer “Champion” was invited to speak about her experiences with cancer and the healthcare system<br><br>- Presentations given by host CBRAs.<br><br>- Well attended. Approximately 40 women attended this event.	
Whitesand First Nation	**July 15, 2013**	**Afternoon presentation**<br><br>- Presentation oriented: Research Team gave slideshow presentation and answered questions.<br><br>- Light refreshments arranged by host CBRA.<br><br>- Approximately 10 people from community attended meeting after being recruited through door-to-door visits.	On-reserve at Community Centre
**City of Thunder Bay**	**Scattered between May 2013 and August 2013**	**Slideshow presentations**<br><br>- BINGO games using cervical cancer vocabulary<br><br>- Poster representation at health fairs and National Aboriginal Day	Our CBRA who targeted urban Aboriginal women organized and was invited to presentations at many different advocacy groups, health centres, and community events, on- and off-reserves.

		- Discussions at prenatal classes and other existing programming	
